# Burden of influenza-associated respiratory hospitalizations in the Americas, 2010–2015

**DOI:** 10.1371/journal.pone.0221479

**Published:** 2019-09-06

**Authors:** Rakhee S. Palekar, Melissa A. Rolfes, C. Sofia Arriola, Belsy O. Acosta, Patricia Alberto Guidos, Xiomara Badilla Vargas, Christina Bancej, Juliana Barbosa Ramirez, Elsa Baumeister, Alfredo Bruno, Maria Agüeda Cabello, Jufu Chen, Paula Couto, Francisco J. De Paula Junior, Rodrigo Fasce, Walquiria Ferreira de Almeida, Victor E. Fiesta Solorzano, Carlos Flores Ramírez, Natalia Goñi, Yadira Isaza de Moltó, Jenny Lara, Diana C. Malo, José L. Medina Osis, Homer Mejía, Lourdes Moreno Castillo, Desiree Mustaquim, Andrea Nwosu, Jenny Ojeda, Antonio Paredes Samoya, Paola A. Pulido, Hector M. Ramos Hernandez, Rudvelinda Rivera Lopez, Angel Rodriguez, Myriam Saboui, Hilda Salazar Bolanos, Adrián Santoro, Jose Eduardo Silvera, Paulina Sosa, Viviana Sotomayor, Lourdes Suarez, Marta Von Horoch, Eduardo Azziz-Baumgartner

**Affiliations:** 1 Pan American Health Organization/World Health Organization, Washington, DC, United States of America; 2 Centers for Disease Control and Prevention, Atlanta, GA, United States of America; 3 Department of Virology, “Pedro Kouri” Institute of Tropical Medicine, Havana, Cuba; 4 Ministry of Health, San Salvador, El Salvador; 5 Costa Rican Department of Social Security, San Jose, Costa Rica; 6 Public Health Agency of Canada, Ottawa, Canada; 7 National Institute of Health, Bogota, Colombia; 8 National Reference Laboratory for Viral Respiratory Infections and National Influenza Center, Buenos Aires, Argentina; 9 National Institute of Public Health Research, Guayaquil, Ecuador; 10 Ministry of Public Health and Social Welfare, Asuncion, Paraguay; 11 Battelle Memorial Institute, Atlanta, GA, United States of America; 12 Ministry of Health, Brasilia, Brazil; 13 Institute of Public Health, Santiago, Chile; 14 National Center of Epidemiology, Prevention, and Control of Diseases, Lima, Peru; 15 Directorate of Health Services, Guatemala City, Guatemala; 16 Department of Public Health Laboratories, Montevideo, Uruguay; 17 Ministry of Health, Panama City, Panama; 18 Costa Rican Institute of Research and Education in Nutrition and Health, Cartago, Costa Rica; 19 Health Secretariat, Tegucigalpa, Honduras; 20 Ministry of Health, Quito, Ecuador; 21 Ministry of Public Health, Guatemala City, Guatemala; 22 Ministry of Health, San Jose, Costa Rica; 23 Directorate of Statistics and Health Information, Buenos Aires, Argentina; 24 Ministry of Health, Montevideo, Uruguay; 25 Ministry of Health, Santiago, Chile; 26 Ministry of Public Health, Havana, Cuba; Ben-Gurion University of the Negev, UNITED STATES

## Abstract

**Background:**

Despite having influenza vaccination policies and programs, countries in the Americas underutilize seasonal influenza vaccine, in part because of insufficient evidence about severe influenza burden. We aimed to estimate the annual burden of influenza-associated respiratory hospitalizations in the Americas.

**Methods:**

Thirty-five countries in the Americas with national influenza surveillance were invited to provide monthly laboratory data and hospital discharges for respiratory illness (International Classification of Diseases 10^th^ edition J codes 0–99) during 2010–2015. In three age-strata (<5, 5–64, and ≥65 years), we estimated the influenza-associated hospitalizations rate by multiplying the monthly number of respiratory hospitalizations by the monthly proportion of influenza-positive samples and dividing by the census population. We used random effects meta-analyses to pool age-group specific rates and extrapolated to countries that did not contribute data, using pooled rates stratified by age group and country characteristics found to be associated with rates.

**Results:**

Sixteen of 35 countries (46%) contributed primary data to the analyses, representing 79% of the America’s population. The average pooled rate of influenza-associated respiratory hospitalization was 90/100,000 population (95% confidence interval 61–132) among children aged <5 years, 21/100,000 population (13–32) among persons aged 5–64 years, and 141/100,000 population (95–211) among persons aged ≥65 years. We estimated the average annual number of influenza-associated respiratory hospitalizations in the Americas to be 772,000 (95% credible interval 716,000–829,000).

**Conclusions:**

Influenza-associated respiratory hospitalizations impose a heavy burden on health systems in the Americas. Countries in the Americas should use this information to justify investments in seasonal influenza vaccination—especially among young children and the elderly.

## Introduction

The majority of countries and territories in the Americas (40 out of 45 countries and territories), have created and implemented seasonal influenza vaccination policies [1]. Although these predominantly high and middle-income countries have such policies, vaccine coverage remains low. For example, during 2009–2015 Peru provided influenza vaccination, free of charge, to older adults but coverage remained low at 26% [2]. Providers are more likely to recommend, and the public are more likely to get, an influenza vaccine when they have information about the risk of severe illness [3, 4], yet surveys suggest that healthcare providers and those targeted for vaccination remain unaware of the risk of severe influenza illness and the benefits of vaccination [5]. Estimates of influenza disease burden are therefore useful for risk communication and for evaluating the national impact of influenza vaccination programs [6]. Few countries in the Americas, however, have published estimates of their annual burden of influenza-associated hospitalizations [7–15].

Estimating the burden of influenza is challenging because few persons with influenza illness are tested and the majority remain undiagnosed [15]. Population-based cohort studies are a source of data for estimating incidence and burden of influenza [2, 16–18], but are expensive and not practical for measuring burden in many countries. An alternate source of data for burden estimation is hospital discharge data combined with influenza virologic surveillance data [19]. This approach assumes that the number of hospitalizations attributable to influenza in a specific age group is proportional to the monthly national-level influenza activity. While there are limitations to this approach, the availability of hospital discharge data and standardized virologic surveillance in the Americas makes it a realistic method to estimate burden in the entire region [20].

We invited countries in the Americas to contribute hospital discharge and national virologic surveillance data to estimate the total annual number of influenza-associated respiratory hospitalizations in the region. Having estimates of influenza-associated morbidity can provide much needed information for effectively communicating the risk and burden of influenza on a population, as well as provide the building blocks for estimates of illness and costs that could be prevented by increasing influenza vaccination coverage.

## Methods

### Data sources

We invited 35 countries in the Americas that participate in SARInet [21] to contribute to this analysis. SARInet is a regional network of hospitals, laboratories, and organizations conducting surveillance for severe acute respiratory infection (SARI) associated with influenza. We asked each country to provide the national number of International Classification of Disease 10^th^ edition (ICD-10) respiratory discharge-coded hospitalizations (J00-99) (primary cause) that occurred monthly during 2010–2015. All respiratory codes were used, as prior analyses have suggested that influenza-associated hospitalizations are better captured using all respiratory codes and using pneumonia and influenza ICD-10 only codes, likely underestimates the respiratory hospitalizations attributable to influenza [22]. Additionally, we asked countries to provide the monthly proportion of samples that tested positive for influenza virus within national-level virologic surveillance platforms, which could consist of specimens from inpatients or outpatients. This decision was based upon the fact that prior analyses that have shown the percent positivity to be only slightly higher among outpatients than inpatients and most countries do not have data stratified by clinical site [7, 17]. We asked that all data be stratified by six age groups, as recommended by the World Health Organization (WHO) for influenza surveillance (<2, 2–4, 5–14, 15–49, 50–64, and ≥65 years) [23], and, if this was not possible, to provide a minimum of three age groups (<5, 5–64, and ≥65 years).

### Calculation of annual influenza-associated hospitalization rates and associated variance among countries providing data

Guidance on calculating annual rates of influenza-associated respiratory hospitalizations was provided to each country [23]. Ten countries provided raw data and six countries provided calculated rates based on the provided guidance. For countries providing raw data, we estimated the monthly number of influenza-associated respiratory hospitalizations, stratified by age group and following the guidance, by multiplying the percent of virologic surveillance specimens testing positive for influenza in a month by the number of respiratory-coded hospitalizations in the same month (S1 Fig). We then summed age-group specific influenza-associated respiratory hospitalizations across a 12-month period to estimate the annual number of influenza-associated respiratory hospitalizations and divided this sum by the age-specific United Nations (UN) population estimate [24] to arrive at an annual rate of influenza-associated respiratory hospitalization. For countries providing hospitalizations from a subset of all of the hospitals within the country (e.g. public hospitals), we adjusted the UN population according to the proportion of the population covered by the hospitals included in the discharge database, as reported by the participating country.

For countries that provided monthly data on hospitalizations and virologic surveillance, we estimated the variance of the monthly number of influenza-associated respiratory hospitalizations assuming that the number of respiratory-coded hospitalizations followed a Poisson distribution and the proportion of samples testing influenza positive followed a binomial distribution. We then summed the monthly variance across each 12-month period (S1 Fig). For countries that provided annual rates, the variance of the rate was provided based on a Poisson distribution. Due to the differences in the data supplied for the analysis, we had to assume that the number of respiratory hospitalizations was independent of the proportion of specimens testing positive for influenza.

### Calculation of pooled rates among countries providing data

Using multi-level random-effects meta-analysis, with clustering by country and log-transformed influenza-associated respiratory hospitalization rates, we pooled the age-stratified hospitalization rates for the years of data provided. We also estimated age-stratified pooled rates by year. We conducted a sensitivity analysis excluding years when <100 specimens in an age group were tested for influenza.

### Building models for extrapolation to countries not providing data

We built meta-regression models to investigate heterogeneity in hospitalization rates between countries and to develop an extrapolate model. A priori, we considered country, year, predominant circulating influenza type/subtype [25], influenza vaccine coverage among target age groups [1], WHO influenza transmission zone [26], World Bank income classification (high, upper-middle, and lower-middle/low) [27], and national hospital bed density (categorized into quartiles of <1.5, 1.5–2.2, 2.3–3.7, ≥3.8 beds/10,000 population) [28] (S1 Table) as potentially important sources of variability in hospitalization rates. Annual influenza vaccine coverage was considered for inclusion; however, data were not available from all the contributing countries using comparable target age groups and, thus, could not assessed as a potential variable for model inclusion. For the indicator of predominant circulating influenza virus, we used virologic data submitted through the Pan-American Health Organization to FluNet, the WHO global platform for influenza virologic surveillance, and classified the predominant circulating virus (either influenza A[H1], influenza A[H3], influenza B, or mixed) based on the influenza virus type/subtype that comprised ≥40% of the annual influenza-positive specimens [25]. Among the countries providing data, we evaluated the association of each of the above variables with the rates using an omnibus test from a bivariate meta-regression model stratified by age group. We did not include a variable for country in final extrapolation models because the objective was to extrapolate beyond contributing countries. Additionally, we did not include year in final models because we wished to estimate the average rate of hospitalization. Aside from these exceptions, we included predictive variables in the final age-group specific extrapolation model if they were associated with hospitalization rates, had a p value ≤0·05 from the omnibus test, and there was sufficient data for the predictive variable from non-contributing countries.

### Estimating annual number of influenza-associated respiratory hospitalizations in the Americas

To estimate the average number of influenza-associated respiratory hospitalizations in the Americas among countries that provided data, we multiplied each country’s age-group specific pooled rate by the average age-group specific UN population estimate from 2010–2015 [24]. To estimate the average number of influenza-associated hospitalizations in countries not providing data, we used the rate from the age-group specific meta-regression extrapolation model and applied this rate to each country’s age-group specific average UN population over the time series. Finally, we summed the estimated counts from all participating and non-participating countries to estimate the average total number of influenza-associated respiratory hospitalizations in the Americas. We rounded all estimates of the total number of hospitalizations to three significant digits. We used Monte Carlo simulations to estimate the 95% credible interval (CrI) of the overall burden. In brief, assuming that pooled and extrapolated rates followed a Poisson distribution, we drew 100,000 samples from each country’s rate distribution and estimated the number of hospitalizations for each of the samples. The 95% CrI were estimated from the 2·5^th^ and 97·5^th^ percentiles of the distributions summed across all countries in the region.

We conducted all analyses in R software version 3·3·2 using the ‘metafor’ package for meta-analysis and meta-regression (version 1·9–9) [29].

## Results

Sixteen out of 35 (46%) SARInet countries contributed data for this analysis; constituting 79% of the total population of the Americas (Table 1). The participating countries represented 25% (5/20) of all high-income countries, 47% (7/15) of all upper-middle income countries, 50% (4/8) of all lower-middle income countries, and no low-income countries (0/1) in the region. Among countries providing data, the median annual number of specimens tested for influenza, as part of the virologic surveillance system, was 866 specimens (interquartile range: 543–3,031; S2 Table); most of the countries (11/16) provided virologic surveillance data from outpatient and inpatient surveillance (S2 Table). The percent of specimens testing positive for influenza varied by year and country ranging from 4·3–9·0% positive among children aged <5 years, 10·0–23·1% among those aged 5–64 years, and 6·8–17·6% among adults aged ≥65 years.

**Table 1:** Crude annual ranges and average pooled estimates of influenza-associated hospitalization rates, per 100,000 people, by age group for 16 countries providing data—2010–2015.

		Aged <5 years			Aged 5–64 years			Aged ≥65 years		
	Years included in analysis	Crude range ^†^, all respiratory hospitalizations	Crude range ^†^, influenza-associated hospitalizations	Pooled Rate ^‡^ (95% CI)	Crude range ^†^, all respiratory hospitalizations	Crude range ^†^, influenza-associated hospitalizations	Pooled Rate ^‡^ (95% CI)	Crude range ^†^, all respiratory hospitalizations	Crude range ^†^, influenza-associated hospitalizations	Pooled Rate ^‡^ (95% CI)
Argentina	2010–2013	1,151–2,471	18–67	34 (15, 75)	239–258	15–36	26 (14, 47)	903–1,073	74–132	94 (47, 188)
Brazil	2010–2015	3,301–4,067	91–273	157 (76, 326)	371–503	23–92	56 (33, 97)	2,948–3,463	47–459	293 (152, 564)
Canada *	2010–2014	1,017–1,832	67–135	113 (53, 241)	203–297	5–12	9 (5, 16)	1,799–2,565	150–677	323 (167, 625)
Chile ^§^	2013, 2014	—	109–206	149 (57, 394)	—	26–61	40 (19, 81)	—	195–241	216 (95, 492)
Colombia	2010–2015	1,202–2,070	37–140	65 (31, 135)	130–215	10–24	15 (9, 26)	590–1,357	30–116	62 (32, 121)
Costa Rica	2010–2014	1,414–1,744	38–201	99 (46, 213)	75–92	4–30	15 (8, 26)	814–949	34–166	88 (45, 173)
Cuba	2011–2015	334–528	13–31	22 (10, 47)	14–20	2–6	3 (2, 5)	128–197	7–46	24 (12, 48)
Ecuador	2012–2015	864–2,111	24–175	70 (31, 155)	237–268	16–57	29 (16, 53)	1,489–1,611	110–219	156 (77, 315)
El Salvador	2010–2013	3,685–5,455	186–383	264 (118, 590)	240–252	34–51	42 (23, 77)	1,819–1,983	229–317	259 (127, 529)
Guatemala	2010–2014	822–1,314	29–547	77 (36, 166)	73–104	6–42	9 (5, 17)	479–814	0–126	55 (16, 198)
Honduras	2010–2014	1,824–2,866	135–359	199 (93, 425)	130–145	16–31	22 (13, 40)	1,527–1,672	81–277	121 (50, 295)
Panama	2011–2013	426–745	8–53	21 (9, 51)	44–51	5–9	7 (3, 13)	1,178–1,336	0–92	92 (27, 320)
Paraguay	2010–2015	1,028–1,601	32–115	60 (29, 125)	93–145	11–27	17 (10, 29)	908–1,148	81–215	108 (55, 211)
Peru	2010–2014	1,719–2,078	103–259	166 (78, 355)	118–147	21–46	29 (16, 51)	1,187–1,383	36–248	137 (69, 271)
United States *	2010–2013	1,222–1,601	49–144	86 (39, 190)	1,135–1,303	46–110	83 (46, 150)	8,727–9,900	287–734	487 (245, 971)
Uruguay	2011–2015	4,731–5,177	181–816	297 (115, 765)	425–571	26–82	59 (30, 114)	2,229–2,852	280–460	383 (170, 862)
**Overall**				**90 (61, 132)**			**21 (13, 32)**			**141 (95, 211)**

* For countries in the North American influenza transmission zone, year of observation was defined from July of the labeled year to June of the subsequent year. For all other countries, year of observation was defined from January to December.

^†^ The crude range of estimated rates for all respiratory hospitalizations, per 100,000 population, and influenza-associated hospitalization, per 100,000.

^‡^ Pooled rates of influenza-associated hospitalizations, per 100,000 population, were estimated using random effects meta-analysis. 95% confidence intervals (CI) were estimated using random effects meta-analysis.

^§^ Chile provided rates of influenza-associated hospitalization, using the same methods, but did not provide the rate of respiratory hospitalization during the study period

The annual rate of respiratory hospitalization by year and country ranged from 334–5,455 per 100,000 in children aged <5 years, 14–1,303 per 100,000 in people aged 5–64 years, and 128–9,900 per 100,000 in adults aged ≥65 years (Table 1). Applying the virologic surveillance data to attribute respiratory hospitalizations to influenza, we observed that crude rates of influenza-associated respiratory hospitalizations varied from 3 per 100,000 in people aged 5–64 years in Cuba to 487 per 100,000 in among people 65+ years in the United States of America (Table 1). From the meta-analysis, the pooled annual rate of influenza-associated respiratory hospitalizations across all contributing countries was 90 per 100,000 (95% confidence interval [CI]: 61–132 per 100,000) among children aged <5 years of age, 21 per 100,000 (95% CI: 13–32 per 100,000) among persons aged 5–64 years, and 141 per 100,000 (95% CI: 95–211 per 100,000) among persons aged ≥65 years. The overall pooled rate among children aged <5 years was 4.5 (95% CI: 3·7–5·4) times the rate among those aged 5–64 years. The rate among adults aged ≥65 years was 6.7 (95% CI: 5·5–8·2) times as high as the rate among those aged 5–64 years. The estimated pooled rates and associations with age were similar when the analysis was restricted to years when countries had tested at least 100 specimens for influenza through their virologic surveillance (results not shown). This exclusion criterion would have resulted in excluding one year in Guatemala from analysis for children aged <5 years, excluding one year in Guatemala from analysis for persons aged 5–64 years, and excluding a total of eleven years from El Salvador (1 year), Guatemala (4 years), Honduras (3 years), Panama (2 years), and Uruguay (2 years) for persons aged ≥65 years.

**Fig 1:**
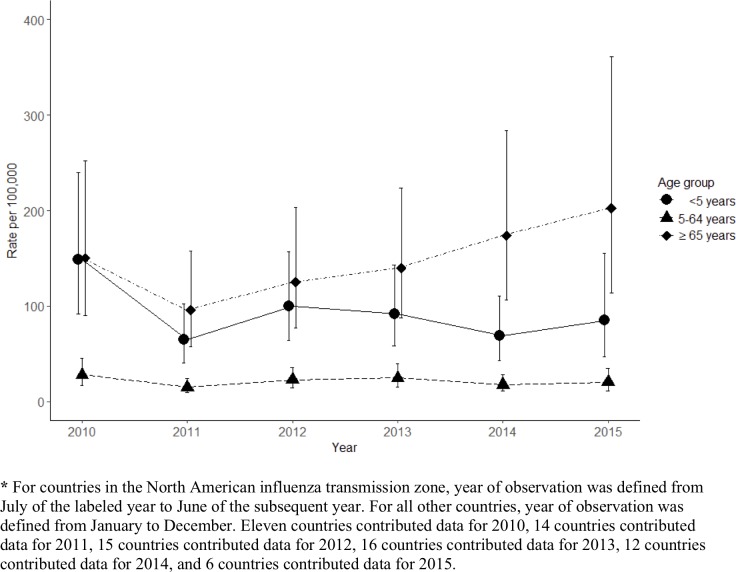
Pooled estimates of influenza-associated hospitalization rates per 100,00 people by year and age group, 2010–2015.

From meta-regression, we found that country and year were both significantly associated with hospitalization rates in all age groups (p<0·001; Table 2). Rates were highest for all years of the analysis, for people aged <65 years (Fig 1). The predominant circulating virus varied year-to-year, however the trend was not consistent across countries. In 2010 influenza A/H1N1pdm09 and A/H3 viruses were equally predominant in participating countries, whereas influenza A/H3 viruses were predominant throughout the region in 2015 (S3 Table). The predominant circulating influenza virus type/subtype was significantly associated with rates of hospitalization from the meta-regression analysis of the 5–64 year age group; rates were significantly higher when influenza A/H1N1pdm09 viruses were predominant than when influenza A/H3N2 was predominant (rate ratio = 1·7, 95% CI: 1·4–2·2; Table 2). This association was also seen for young children, aged <5 years (rate ratio = 1.6, 95% CI: 1·1–2·2), but no association was seen between predominant virus type/subtype and hospitalization rates in adults aged ≥65 years. Virologic predominance data, however, were not available from the majority of countries we were extrapolating to, thus we were unable to include viral predominance in extrapolation models. In older adults, aged ≥65 years, and young children, countries with a greater density of hospital beds appeared to have higher hospitalization rates (Table 2). This trend was not monotonic across density quartiles and was only statistically significant for rates among adults aged ≥65 years; though there were significantly higher rates in young children, aged <5 years, in the upper three quartiles when each was compared with the lowest quartile. The variability of rates in older adults were also, to a certain extent, explained by the income status of the country (p = 0·06) and the WHO influenza transmission zone (p = 0·07).

**Table 2:** Bivariate association between influenza-associated respiratory hospitalization rates and predictive covariates among 16 countries in the Americas participating in the analysis, 2010–2015.

** **	<5 years		5–64 years		≥65 years	
	Rate ratio * (95% CI)	P-value ^†^	Rate ratio * (95% CI)	P-value ^†^	Rate ratio * (95% CI)	P-value ^†^
Country	—	<0·001	—	<0·001	—	<0·001
Year ^‡^		0·002		<0·001		0·025
2010	*Reference*		*Reference*		*Ref Reference*	
2011	0·4 (0·3, 0·7)		0·5 (0·4, 0·7)		0·6 (0·4, 1·0)	
2012	0·7 (0·5, 1·0)		0·8 (0·6, 1·1)		0·8 (0·6, 1·2)	
2013	0·6 (0·4, 0·9)		0·9 (0·7, 1·2)		0·9 (0·6, 1·4)	
2014	0·5 (0·3, 0·7)		0·6 (0·5, 0·9)		1·1 (0·7, 1·7)	
2015	0·6 (0·3, 1·0)		0·7 (0·5, 1·1)		1·3 (0·8, 2·2)	
WHO influenza transmission zone		0·96		0·21		0·07
North America	*Reference*		*Reference*		*Reference*	
Central American Caribbean	0·8 (0·2, 3·1)		0·4 (0·1, 1·7)		0·2 (0·1, 0·7)	
Tropical South America	1·1 (0·2, 4·6)		1·1 (0·3, 4·5)		0·4 (0·1, 1·2)	
Temperate South America	1·0 (0·2, 4·2)		1·2 (0·3, 4·9)		0.4 (0.1, 1·4)	
Predominant virus ^§^		0·06		<0·001		0·31
A/H3 viruses	*Reference*		*Reference*		*Reference*	
A/H1 viruses	1·6 (1·1, 2·2)		1·7 (1.4, 2.2)		1·1 (0·8, 1·5)	
B viruses	1·2 (0·7, 1·9)		1·2 (0·8, 1·6)		0·7 (0·4, 1·1)	
Mixed	1·4 (0·9, 2·3)		1·3 (0·9, 1·8)		1·1 (0·7, 1·7)	
World Bank income category		0·19		0·31		0·06
Low-middle/Low	*Reference*		*Reference*		*Reference*	
Upper-middle	0·4 (0·2, 1·1)		0·8 (0·2, 2·4)		0·7 (0·3, 2·0)	
High	0·7 (0·2, 2·0)		1·6 (0·5, 5·8)		1·9 (0·6, 5·8)	
Density of hospital beds, per 10,000 (in quartiles)		0·08		0·43		<0·001
<1·5	*Reference*		*Reference*		*Reference*	
1·5–2·2	3·6 (1·2, 11·3)		3·2 (0·7, 13·8)		6·5 (2·7, 16·1)	
2·3–3·7	3·6 (1·1, 12·0)		3·0 (0·7, 13·8)		2·6 (1·1, 6·7)	
≥3·8	4.3 (1·4, 13·4)		2·1 (0·5, 9·1)		2·5 (1·0, 6·3)	

* Rate ratios were estimated from a bivariate random effects meta-regression of log-transformed rates of influenza-associated hospitalization and the specified covariate; the rate ratios compare the rate in the category to the reference. A rate ratio is provided because rates were log-transformed prior to analysis in the meta-regression.

^†^ P-values from an omnibus test of association between the log-transformed rate of influenza-associated hospitalization and the specified covariate in a bivariate random effects meta-regression.

^‡^ For countries in the North American influenza transmission zone, year of observation was defined from July of the labeled year to June of the subsequent year. For all other countries, year of observation was defined from January to December. Eleven countries contributed data for 2010, 14 countries contributed data for 2011, 15 countries contributed data for 2012, 16 countries contributed data for 2013, 12 countries contributed data for 2014, and 6 countries contributed data for 2015.

^§^ Predominant virus was determined based on the influenza virus type/subtype was comprised ≥40% of the annual influenza-positive specimens submitted to national virologic surveillance. Mixed predominance occurred when no single virus type/subtype comprised ≥40% of specimens.

**Fig 2:**
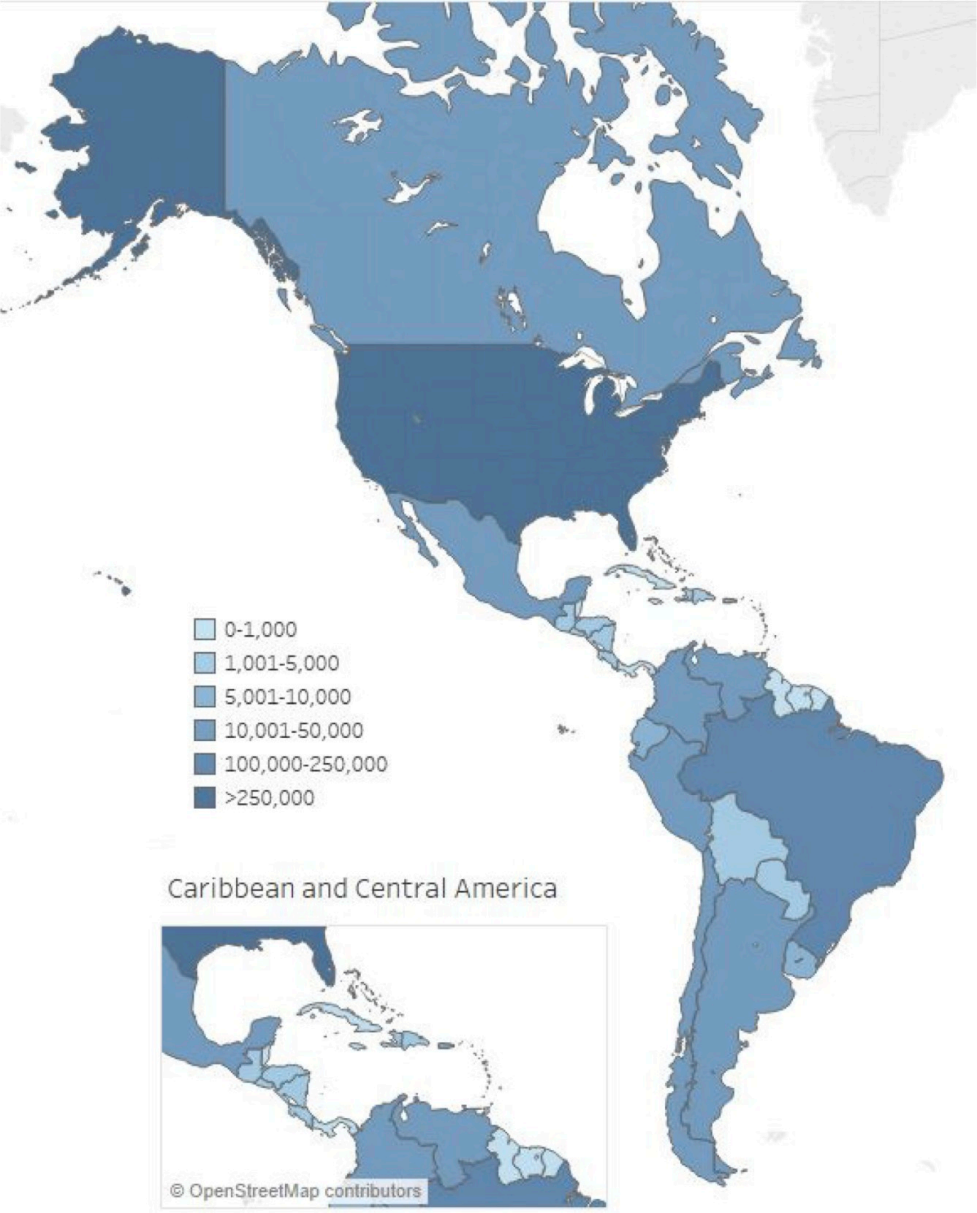
Estimated annual number of influenza-associated respiratory hospitalizations by country in the Americas.

As no overall associations were seen in the <5 and 5–64 year age groups between the country characteristics we explored and the estimated influenza-associated respiratory hospitalization rates, we used the crude rate in these age groups to extrapolate to the countries that did not provide data. For adults aged ≥65 years, we used a model that included the pooled rate plus the density of hospital beds. With these models, we estimated that 772,000 (95% credible interval [CrI]: 716,000–829,000) influenza-associated respiratory hospitalizations occur annually in the region (Fig 2). This included 78,300 (95% CrI: 72,700–84,200) influenza-associated respiratory hospitalizations each year among young children aged <5 years, 384,000 (95% CrI: 332,000–437,000) hospitalizations among those aged 5–64 years, and 310,000 (95% CrI: 291,000–330,000) hospitalizations among adults aged ≥65 years.

## Discussion

Using data from 16 countries with 79% of the population in the Americas, we estimated an average of 772,000 respiratory hospitalizations attributed to influenza occur each year throughout the region. Estimated rates of influenza-associated respiratory hospitalizations were highest among the elderly and young children.

For some countries in the region, this is the first estimate of the burden of seasonal influenza. These estimates can serve as the basis for many public health actions including communicating the risk of seasonal influenza to the public and health care providers, estimating and projecting resource needs in hospitals during seasonal epidemics and pandemics, and building and maintaining evidence for sustained investment in seasonal influenza vaccination. Influenza vaccine remains one of our best tools for preventing influenza and estimates of the burden of influenza can be used to target vaccination campaigns to specific subpopulations, but also as a basis for evaluating a vaccination program [6]. By estimating or using known patterns of healthcare utilization, our estimates of the burden of hospitalizations related to influenza could also be expanded to estimate the burden of influenza on outpatient clinics and in the community [6, 12].

We observed differences in the burden of influenza within the region possibly arising from differences in the way that hospitalizations are captured by a country or the strength of a country’s influenza virologic surveillance system, but also potentially due to characteristics such as a country’s income and hospital bed density or influenza-specific characteristics such as predominant circulating virus and transmission zone. When we statistically explored specific characteristics of a country that could be related to differences in rates, few were predictive in all age groups except country and year. This suggests that there are factors intrinsic to a country that we were unable to measure that might be important in describing the variation in hospitalization burden such as local influenza clade predominance, health-care system structure, health-care seeking behavior, hospital admission practices, and surveillance methodology, among others [8, 30]. Influenza vaccination coverage might also explain some of the variability in hospitalization rates and we wished to explore this further; however, we found that there are differences in how vaccination coverage is estimated in each country and target age groups differed enough between countries that we were not able to directly compare the currently available coverage data [1].

We did find that, in older adults, the hospital bed density was a significant factor in the variability in hospitalization rates—countries with greater hospital bed capacity had higher rates of hospitalization. Hospital bed density might serve as a proxy for access to health-care for older patients and it logically follows that demand, or use, of a hospital system would increase as the supply of hospital beds increases. Furthermore, we saw that influenza-associated respiratory hospitalization rates among the elderly were the highest in high-income countries, especially Canada and the United States, and lower among other countries (e.g. Costa Rica, Cuba, Ecuador, and Guatemala). These differences may be due, in part, to national variation in disease severity stemming from differences in the underlying prevalence of medical conditions, and influenza vaccination coverage or differences in hospital utilization, care-seeking behavior, and clinical practices, including influenza testing and submission of specimens to the national surveillance system [2, 31–33]. There is some evidence that the differences in hospital rates may not reflect true differences in disease severity as the estimates of influenza-associated mortality in Argentina, for example, are substantially higher than in Canada or the United States [34, 35]. We observed higher rates of hospitalization during influenza A/H1-predominant years among those under 65 years of age, a finding which has been shown in limited other analyses in this region [36] and deserves further exploration in future studies, especially taking into account vaccine immunogenicity and vaccine mismatch. This finding might be explained by a selection bias in the surveillance system toward non-elderly patients and health-seeking patterns among the non-elderly, especially in the ambulatory setting, that could increase the percent positivity for influenza among this age group and hence the hospitalization rate.

The estimates generated from our analyses are were similar to those from an active surveillance cohort in Peru (60 hospitalizations per 100,000 adults aged ≥65 years) [2], but lower than rates from a prospective pediatric cohort study in Argentina (200 hospitalizations per 100,000 children aged <5 years) [17]. The cohort in Argentina overlapped with the 2009 H1N1 pandemic, which may have increased incidence of influenza among children. Conversely, our results were similar to modeled estimates among older adults aged ≥65 years from Argentina and the United States, despite our models using different data and methodologies. In Argentina, time-series based models and influenza-associated circulatory and respiratory hospitalizations were used to estimate excess hospitalizations due to influenza [7], whereas estimates from the United States came from mathematical multipliers and population-based influenza inpatient surveillance [12]. Additionally, our estimates among young children, aged <5 years, were also comparable to a recent analysis of influenza-associated acute lower respiratory infection hospitalizations in the Americas [19] and are comparable to an analysis using influenza-associated severe pneumonia rates in El Salvador [8]; both studies used similar methods to this analysis, attributing rates of hospitalization to influenza based on virologic surveillance.

Several countries, including Argentina, Chile, and many Central American countries, have used virologic surveillance data to attribute hospitalizations to influenza, as we did in this analysis, but used a smaller subset of “pneumonia and influenza” ICD-10 hospital discharge codes, rather than all respiratory hospitalizations [7, 10, 36]. Using “pneumonia and influenza” hospitalizations likely under-estimated the true respiratory hospitalization burden from influenza, because patients with influenza can present with a wide spectrum of clinical respiratory symptoms, including exacerbations of chronic respiratory conditions [22, 37–40]. Our approach of using all respiratory codes, therefore, generated higher rates compared to the published rates using only “pneumonia and influenza” codes and are likely more inclusive and reflective of the full breadth of conditions seen in patients with influenza. In countries outside of the Americas, influenza-associated hospitalization rates have been estimated using SARI surveillance and these rates, in Madagascar for example, are comparable to our rates for children less than five years of age (128/1000,000) but were lower for those 65+ years (55/100,000); while in Rwanda, are higher for children less than five years of age (169/10,000) but lower for those 65+ years (34/100,000) [41, 42]. These slight differences in rates might be attributable to variable health-seeking behavior in these countries, as compared to the Americas as a whole but importantly, the rates in these countries are comparable to the rates we observed among individual countries in our analysis.

This analysis is subject to several limitations. First, hospital coding practices, clinical and social thresholds for hospitalization, criteria for laboratory testing, use of influenza diagnostic tests with lower sensitivity, and health-seeking behavior patterns, could influence estimates of influenza-associated hospitalizations and such differences between countries is not fully accounted for using our methods [2, 17, 33, 43, 44]. Second, for the majority of countries in Latin America, active virologic surveillance is carried out mostly among the pediatric population (accounting for >50% of specimens) [45]. As there were fewer virologic data in those aged ≥5 years, our estimates in these age groups are less precise. Third, we assumed that reporting was complete for all hospitals contributing data to the national discharge system; however, underreporting from hospitals is possible, which would have led to under-estimation of the rates and may explain some of the variability between countries. Fourth, most countries contributed hospitalization counts from public rather than private hospitals. While we did account for the proportion of the total population likely covered by public hospitals, we recognize that obtaining data from a subset of hospitals means that our estimates might not be representative of all hospitalizations in a county. Next, for some countries, the virologic surveillance data could not be disaggregated between inpatient and outpatient samples, and the influenza percent positivity might differ in these two settings [46–49]. Next, our methodologic approach was simple and because it was used by all countries, allowed us to directly compare rates within the region. One of its strengths is the ability to easily integrate influenza surveillance data, which will lead to more robust and reliable estimates as surveillance systems improve and expand. However, this methodological approach might not be the best approach for each country, given some of the nuances in influenza virologic surveillance and hospital discharge data already discussed. Thus, country-specific estimates shown here may differ from estimates that a country has published elsewhere using a different methodology. Other approaches such as time series analyses (e.g. Serfling method) have been used [50–53]; however, these ecological models are unable to estimate the attributable excess of hospitalizations due to influenza, as they do not incorporate individual exposure data. Finally, we were unable to account for possible correlation between the number of respiratory hospitalizations and the proportion of specimens testing positive for influenza, which may make our confidence intervals too tight.

In conclusion, we estimated an important burden of influenza-associated respiratory hospitalizations in the Americas using routinely collected and timely data from the majority of the population in the region. Future analyses can expand upon our analysis and focus on using these burden data to estimate the economic burden of influenza disease, the impact of current influenza vaccination, and the potential benefits of increasing vaccination coverage particularly among children and older adults where the burden was greatest. Such estimates can help countries better understand the value of and guide investment in seasonal influenza vaccination programs.

## Supporting information

S1 Fig Methods for calculating country- and age-specific crude annual rates of influenza-associated respiratory hospitalizations, per 100,000. (DOCX)

S1 Table Density of hospital beds and World Bank income classification for countries in the Americas. (DOCX)

S2 Table Data sources and descriptions from countries contributing data to the estimate of influenza-associated respiratory hospitalizations in the Americas. (DOCX)

S3 Table Summary of predominant circulating influenza virus among contributing countries by WHO influenza transmission zone, 2010–2015. (DOCX)
