# Supplementary material for: Burden of influenza-associated respiratory hospitalizations in the Americas, 2010–2015
Source: PLoS One. 2019 Sep 6;14(9):e0221479. doi: 10.1371/journal.pone.0221479 (PMC6730873; doi:10.1371/journal.pone.0221479)
Supplement: S1 Fig — (DOCX) [file pone.0221479.s001.docx]

**S1 Fig: Methods for calculating country- and age-specific crude annual rates of influenza-associated respiratory hospitalizations, per 100,000**
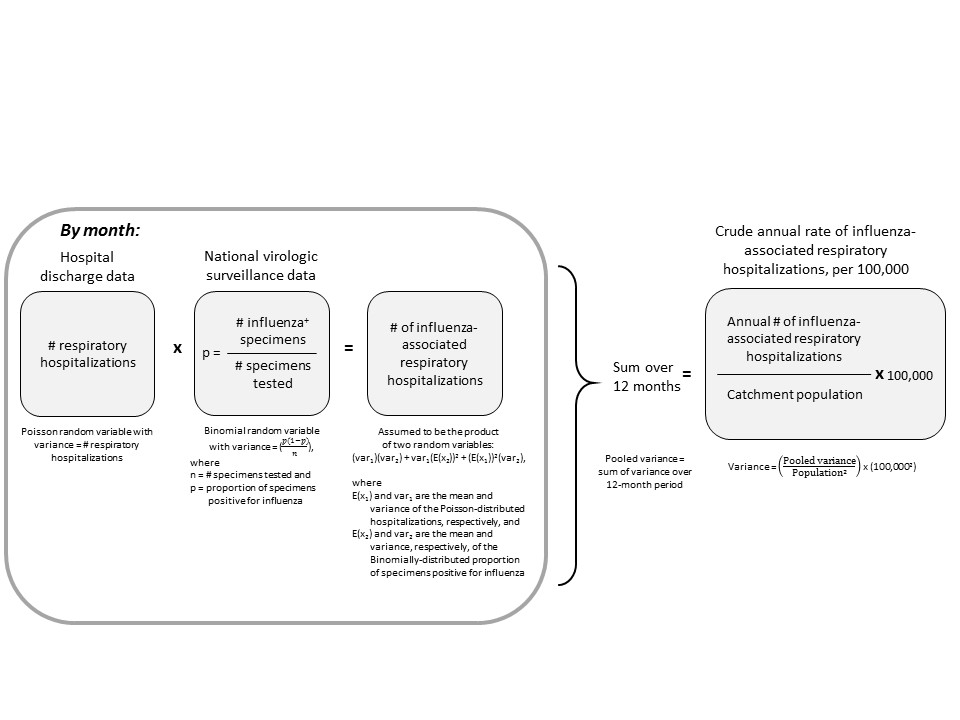


Ten countries provided monthly counts of hospital discharge data and monthly data on the number of specimens tested and the proportion positive for influenza. For these countries, we were able to estimate monthly variance for the number of influenza-associated respiratory hospitalizations and evaluate whether the hospital count was independent of, i.e. not correlated with, the proportion of specimen testing influenza positive. Using monthly data, we observed that most countries did exhibit some degree of non-zero correlation, though there were no obvious patterns across countries or age groups (median correlation [min, max] across all years for kids aged <5 years: 0.08 [-0·75, 0·95]; those aged 5–64 years: 0·56 [-0·64, 0·91]; and adults aged ≥65 years: 0·49 [-0·57, 0·92]).

However, six countries only provided annual rates of influenza-associated respiratory hospitalizations, which was insufficient to include an estimate of covariance in the variance of the rate. Thus, we decided to assume independence between the number of respiratory hospitalizations and the proportion of specimens testing influenza positive for all 16 countries. We recognize the main limitation of this assumption is that we are under-estimating the variability of the annual rate estimates and this yields tighter credible intervals.
