# Supplementary material for: Burden of influenza-associated respiratory hospitalizations in the Americas, 2010–2015
Source: PLoS One. 2019 Sep 6;14(9):e0221479. doi: 10.1371/journal.pone.0221479 (PMC6730873; doi:10.1371/journal.pone.0221479)
Supplement: S1 Table — (DOCX) [file pone.0221479.s002.docx]

**S1 Table: Density of hospital beds and World Bank income classification for countries in the Americas**

| **Country** | **Density of hospital beds, per 10,000** | **World Bank income classification** |
| --- | --- | --- |
| Anguilla | 2·3 | High |
| Antigua and Barbuda | 3·9 | High |
| Argentina | 4·9 | High |
| Aruba | 2·8 | High |
| Bahamas | 2·9 | High |
| Barbados | 6·2 | High |
| Belize | 0·9 | Lower Middle |
| Bermuda | 5·2 | High |
| Bolivia | 1·1 | Lower Middle |
| Brazil | 2·3 | Upper Middle |
| British Virgin Islands | 1·7 | High |
| Canada | 2·7 | High |
| Cayman Islands | 2·5 | High |
| Chile | 2·2 | High |
| Colombia | 1·5 | Upper Middle |
| Costa Rica | 1·1 | Upper Middle |
| Cuba | 5·1 | Upper Middle |
| Curacao | 4·2 | High |
| Dominica | 3·8 | Upper Middle |
| Dominican Republic | 1·6 | Upper Middle |
| Ecuador | 1·5 | Lower Middle |
| El Salvador | 1·1 | Lower Middle |
| French Guiana | 2·6 | *Unavailable* |
| Grenada | 3·7 | Upper Middle |
| Guadeloupe | 5·2 | *Unavailable* |
| Guatemala | 0·6 | Lower Middle |
| Guyana | 2·0 | Lower Middle |
| Haiti | 0·7 | Low |
| Honduras | 0.7 | Lower Middle |
| Jamaica | 1·7 | Upper Middle |
| Martinique | 4·1 | *Unavailable* |
| Mexico | 1·5 | Upper Middle |
| Montserrat | 5·8 | *Unavailable* |
| Netherland Antilles | *Unavailable* | High |
| Nicaragua | 0·9 | Lower Middle |
| Panama | 2·3 | Upper Middle |
| Paraguay | 1·3 | Upper Middle |
| Peru | 1·5 | Upper Middle |
| Puerto Rico | 3·4 | High |
| Saint Kitts and Nevis | 4·7 | High |
| Saint Lucia | 1·6 | Upper Middle |
| Saint Martin | 2·0 | *Unavailable* |
| Saint Vincent and the Grenadines | 2·5 | Upper Middle |
| Suriname | 3·1 | Upper Middle |
| Trinidad and Tobago | 2·7 | High |
| Turks and Caicos | 0·6 | High |
| United States of America | 2·9 | High |
| Uruguay | 2·5 | High |
| Venezuela | 0·9 | High |
