# Supplementary material for: Burden of influenza-associated respiratory hospitalizations in the Americas, 2010–2015
Source: PLoS One. 2019 Sep 6;14(9):e0221479. doi: 10.1371/journal.pone.0221479 (PMC6730873; doi:10.1371/journal.pone.0221479)
Supplement: S2 Table — (DOCX) [file pone.0221479.s003.docx]

**S2 Table: Data sources and descriptions from countries contributing data to the estimate of influenza-associated respiratory hospitalizations—Americas**

| **Country** | **WHO influenza transmission zone** | **World Bank income classification** | **Density of hospital beds, per 10,000** | **Years included** | **Source of hospital discharge codes** | **Source of virologic data** | **Diagnostics used** | **Median annual samples tested (range)** | **Among children aged**  **<5 years** | | **Among adults aged**  **≥65 years** | |
| --- | --- | --- | --- | --- | --- | --- | --- | --- | --- | --- | --- | --- |
|  |  |  |  |  |  |  |  |  | **% of samples tested** | **% of respiratory hospitalizations** | **% of samples tested** | **% of respiratory hospitalizations** |
| Argentina | Temperate South America | High income | 4·9 | 2010–2013 | -- All hospitals -- Catchment area is 100% of population | Inpatient and outpatient specimens | RT-PCR and immunofluorescence assay | 8,032 (1,942-51,212) | 84% | 34% | 2% | 22% |
| Brazil | Tropical South America | Upper middle income | 2·3 | 2010–2015 | -- All federally-supported hospitals -- Catchment area is 75% of population -- Primary discharge diagnoses considered | Inpatient specimens | RT-PCR and immunofluorescence assay; 10% of negatives by immunofluorescence assay are re-tested with RT-PCR | 4,378 (1,955-6,463 | 39% | 32% | 12% | 26% |
| Canada | North America | High income | 2·7 | 2010–2014 | -- All public hospitals -- One province excluded -- Catchment area is 100% of included provinces | Inpatient and outpatient specimens | RT-PCR | 19,919 (16,816-64,852) | 15% | 15% | 13% | 50% |
| Chile ^a^ | Temperate South America | High income | 2·2 | 2013–2014 | -- All public and private hospitals -- Catchment area is 100% of population -- Primary discharge diagnosis considered | Inpatient specimens | RT-PCR and immunofluorescence assay |  |  |  |  |  |
| Colombia | Tropical South America | Upper middle income | 1·5 | 2010–2015 | -- All public and private hospitals -- Catchment area is 100% of population -- Primary discharge diagnosis considered | Inpatient and outpatient specimens | 40% tested by immunofluorescence assay with 5% of negatives re-tested by RT-PCR; 60% tested with RT-PCR | 1,685 (821-2,877) | 44% | 35% | 11% | 17% |
| Costa Rica | Central American Caribbean | Upper middle income | 1·1 | 2010–2014 | -- All public hospitals -- Catchment area is 87% of population | Inpatient and outpatient specimens | 20% tested by immunofluorescence assay; 80% tested by RT-PCR | 798 (521-1,085) | 47% | 46% | 17% | 27% |
| Cuba | Central American Caribbean | Upper middle income | 5·1 | 2011–2015 | -- All hospitals -- Catchment area is 100% of population | Inpatient specimens | Immunofluorescence assay | 1,399 (465-1,853) | 44% | 40% | 9% | 36% |
| Ecuador | Tropical South America | Lower middle income | 1·5 | 2012–2015 | -- Catchment area is 100% of population | Inpatient specimens | RT-PCR | 846 (561-2,533) | 72% | 36% | 9% | 22% |
| El Salvador | Central American Caribbean | Lower middle income | 1·1 | 2010–2013 | -- All public hospitals -- Catchment area is 80% of population | Inpatient and outpatient specimens | 20% tested by immunofluorescence assay; 80% tested by RT-PCR | 454 (258-1,021) | 65% | 57% | 9% | 18% |
| Guatemala | Central American Caribbean | Lower middle income | 0·6 | 2010–2014 | -- All public hospitals -- Catchment area is 70% of population | Inpatient and outpatient specimens | 20% tested by immunofluorescence assay; 80% tested by RT-PCR | 102 (33-348) | 75% | 59% | 5% | 15% |
| Honduras | Central American Caribbean | Lower middle income | 0·7 | 2011–2014 | -- All public hospitals -- Catchment area is 60% of population | Inpatient and outpatient specimens | 20% tested by immunofluorescence assay; 80% tested by RT-PCR | 632 (105-1,135) | 60% | 64% | 4% | 14% |
| Panama | Central American Caribbean | Upper middle income | 2·3 | 2011–2013 | -- All public hospitals -- Catchment area is 40% of population | Inpatient and outpatient specimens | 20% tested by immunofluorescence assay; 80% tested by RT-PCR | 440 (102-1,570) | 80% | 31% | 2% | 48% |
| Paraguay | Temperate South America | Upper middle income | 1·3 | 2010–2015 | --All public and social security hospitals  --Catchment area is 89% of population  --Primary discharge diagnosis considered | Inpatient specimens | RT-PCR and immunofluorescence assay | 866 (552-1,430) | 60% | 47% | 12% | 19% |
| Peru | Tropical South America | Upper middle income | 1·5 | 2010–2014 | -- Public hospitals -- Catchment area is 48% of population -- Primary discharge diagnosis considered | Inpatient and outpatient specimens | RT-PCR and immunofluorescence assay | 1,599 (666-2,038) | 43% | 49% | 10% | 21% |
| United States | North America | High income | 2·9 | 2010–2013 | -- Nationwide database of healthcare encounters (MarketScan) -- Catchment area is 8% of population -- All discharge diagnoses considered | Inpatient and outpatient specimens | Any approved diagnostic technique | 38,069 (32,115-69,127) | 20% | 4% | 21% | 40% |
| Uruguay | Temperate South America | High income | 2·5 | 2011–2015 | -- All hospitals -- Catchment area is 100% of population | Inpatient and outpatient specimens | RT-PCR | 160 (112-344) | 41% | 31% | 21% | 32% |

^a^ Chile provided rates of influenza-associated hospitalization, using the same methods, but did not provide the rates of respiratory hospitalization or virologic surveillance information directly
