# Supplementary material for: Burden of influenza-associated respiratory hospitalizations in the Americas, 2010–2015
Source: PLoS One. 2019 Sep 6;14(9):e0221479. doi: 10.1371/journal.pone.0221479 (PMC6730873; doi:10.1371/journal.pone.0221479)
Supplement: S3 Table — (DOCX) [file pone.0221479.s004.docx]

**S3 Table: Summary of predominant circulating influenza virus among contributing countries by WHO influenza transmission zone, 2010–2015 ^a^**

| **North America** | **2010** | **2011** | **2012** | **2013** | **2014** | **2015** |
| --- | --- | --- | --- | --- | --- | --- |
| Canada | H3 | B | H3 | H1/B | H3 |  |
| United States | H3 | H3 | H3 | H1 |  |  |
| **Central America/Caribbean** |  |  |  |  |  |  |
| Costa Rica | H1 | H3 | H3/B | H1 | H3/B |  |
| Cuba |  | H3 | B | H1 | H3/B | H1 |
| El Salvador | + | H3/B | H1/B | H1/H3 |  |  |
| Guatemala | H1 | H3 | H1 | H3 | + |  |
| Honduras | H3 | H3 | H1/H3 | H1 | B |  |
| Panama |  | + | B | H3 |  |  |
| **Tropical South America** |  |  |  |  |  |  |
| Brazil | H1 | H3 | H1/H3 | H1 | H3 | H3 |
| Colombia | H1 | H1 | H3 | H1 | H3 | H3 |
| Ecuador |  |  | H3/B | H1 | B | H3 |
| Peru | H1/H3 | H3 | B | H1 | H3 |  |
| **Temperate South America** |  |  |  |  |  |  |
| Argentina | B | H3 | B | H1 |  |  |
| Chile |  |  |  | H1 | H3 |  |
| Paraguay | H3 | H3 | H1 | H3 | H3 | H3 |
| Uruguay |  | H1 | + | H1 | + | + |

^a^ We determined predominant virus based on the influenza virus type/subtype was comprised ≥40% of the annual influenza-positive specimens submitted to national virologic surveillance. We only determined the predominant virus for the years of burden data that were provided. We defined the year from July through June for countries in North America and as a calendar year for all other countries. Eleven countries contributed data for 2010, 14 countries contributed data for 2011, 15 countries contributed data for 2012, 16 countries contributed data for 2013, 12 countries contributed data for 2014, and 6 countries contributed data for 2015.

^+^ Virologic data were available, however the total number of influenza-positive samples was <100 for that year.
